# Primary Thyroid Lymphoma: A Report of Three Cases and a Review of the Literature

**DOI:** 10.7759/cureus.103657

**Published:** 2026-02-15

**Authors:** Chaimae Daoudi, Nassira Karich, Imane Demnati, Anass Haloui, Amal Bennani

**Affiliations:** 1 Department of Pathology, Mohammed VI University Hospital, Faculty of Medicine, Mohammed 1st University, Oujda, MAR; 2 Department of Otorhinolaryngology, Mohammed VI University Hospital, Faculty of Medicine, Mohammed 1st University, Oujda, MAR

**Keywords:** cervical mass, diffuse large b-cell lymphoma, hashimoto’s thyroiditis, malt lymphoma, primary thyroid lymphoma

## Abstract

Primary thyroid lymphoma is an uncommon malignant tumor of the thyroid gland. The most frequent histological subtype is diffuse large B-cell lymphoma, which typically occurs in middle-aged to elderly women and presents as a rapidly enlarging cervical mass. We report the cases of three women, aged between 50 and 75 years, who presented with rapidly enlarging cervical masses accompanied by compression symptoms such as dyspnea and dysphagia. Cervicothoracic computed tomography (CT) scans revealed a plunging goiter in two patients and a well-defined thyroid mass in the third. Surgical biopsies were conducted on all three patients, and histological examination, coupled with immunohistochemistry, confirmed B-cell lymphomatous proliferation.

## Introduction

Primary thyroid lymphoma (PTL) is defined as a lymphoma that originates primarily from the thyroid gland, rather than secondary involvement resulting from dissemination from another primary site [1]. PTL is a rare malignancy, accounting for approximately 1-5% of all malignant thyroid tumors and 1-2% of extranodal lymphomas [2].

This entity predominantly affects women, with a reported female-to-male ratio of approximately 8:1 [3]. PTL typically occurs in the sixth to seventh decades of life, with a markedly lower incidence in patients younger than 40 years [2,3].

Histologically, PTL most commonly presents as a non-Hodgkin lymphoma, with diffuse large B-cell lymphoma (DLBCL) representing the most frequent subtype, accounting for 50-70% of cases [4]. This is followed by extranodal marginal zone B-cell lymphoma of mucosa-associated lymphoid tissue (MALT) (20-30%), follicular lymphoma (approximately 12%), Hodgkin lymphoma (7%), small lymphocytic lymphoma (4%), and Burkitt lymphoma (4%) [5].

Primary T-cell lymphomas of the thyroid are exceedingly rare and pose significant diagnostic challenges [2].

PLT represents a major diagnostic challenge because of its close differential diagnosis with anaplastic thyroid carcinoma; therefore, histopathological examination combined with immunohistochemical analysis is essential for establishing an accurate diagnosis.

## Case presentation

Case 1

**Figure 1:**
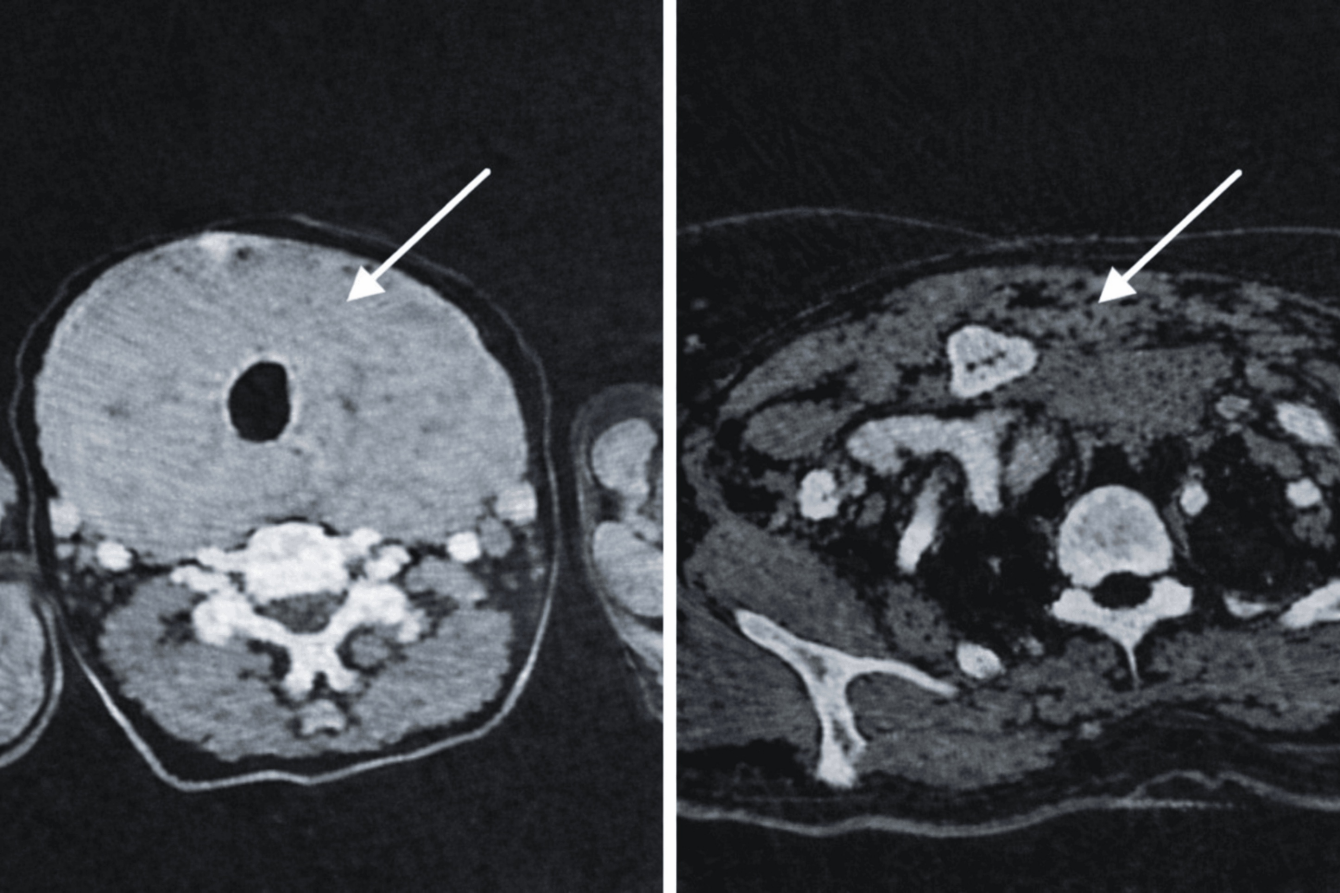
Axial cervicothoracic computed tomography scan demonstrating a voluminous plunging goiter arising from the thyroid gland and extending into the mediastinum White arrows indicate the location of the goiter in the cervical and mediastinal regions, showing its extension and mass effect on surrounding structures.

A 50-year-old female patient with no significant past medical history presented with cervical swelling that had been present for 6 months and was associated with dysphagia and dyspnea. On clinical examination, the patient was conscious with stable hemodynamic and respiratory status. Cervical examination revealed a painful cervical swelling on palpation that was mobile during swallowing. Laboratory evaluation showed a thyroid-stimulating hormone level of 8.71 mIU/L, free thyroxine of 8.64 mIU/L, and free triiodothyronine of 3.68 mIU/L, consistent with peripheral hypothyroidism. Anti-thyroid peroxidase antibodies were elevated at 177.88 IU/mL. Cervical ultrasonography demonstrated a diffuse heterogeneous goiter associated with right supraclavicular lymphadenopathy measuring 20 × 10 mm. Cervicothoracic CT revealed a heterogeneous plunging goiter without suspicious lesions (Figure 1).

A tracheotomy with surgical biopsy of the mass was performed.

**Figure 2:**
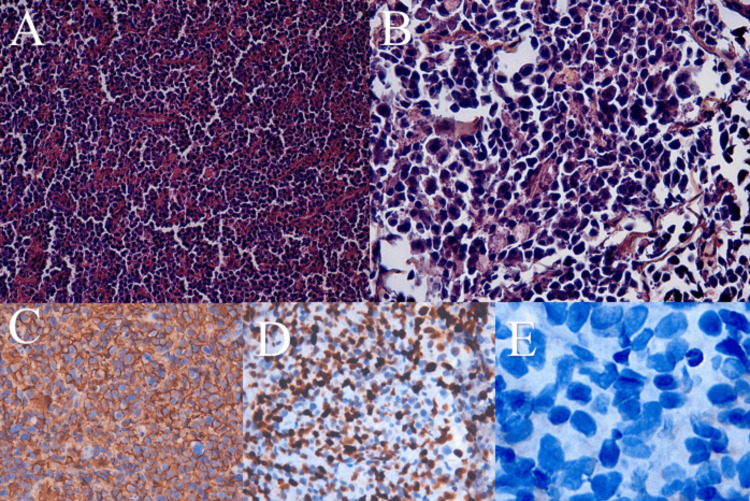
Histopathological and immunohistochemical findings (Case 1) (A) Histopathological section showing tumor cells arranged in diffuse sheets (hematoxylin and eosin stain, ×10). (B) Higher-power view showing large, atypical tumor cells with round nuclei, clear-appearing cytoplasm, hyperchromatic nuclei, and numerous mitotic figures (hematoxylin and eosin stain, ×40). (C) Strong and diffuse CD20 positivity in tumor cells (immunohistochemistry). (D) High tumor proliferation index, estimated at approximately 85% (Ki-67 immunostaining). (E) Absence of tumor cell staining for cytokeratin (immunohistochemistry).

Histological examination revealed a malignant tumor proliferation arranged predominantly in diffuse sheets. The tumor cells were dyscohesive, large, and atypical, with hyperchromatic nuclei and clear-appearing cytoplasm. Numerous mitotic figures were identified (Figures 2A, 2B).

Immunohistochemical analysis demonstrated strong and diffuse positivity of tumor cells for CD20 (Figure 2C), a high tumor proliferation index estimated at approximately 85% (Figure 2D), and absence of tumor cell staining for cytokeratin and CD3 (Figure 2E). Tumor cells were also negative for CD10 and BCL6.

Based on the combined histological and immunohistochemical findings, a diagnosis of diffuse large B-cell lymphoma of the thyroid was established.

The patient initiated chemotherapy treatment following the R-CHOP (Rituximab, Cyclophosphamide, Doxorubicin, Vincristine, and oral Prednisolone) protocol, resulting in rapid regression of the mass, but unfortunately, the patient subsequently passed away.

Case 2

A 75-year-old woman with a history of hypertension was admitted for cervical swelling. On clinical examination, the patient was conscious with stable hemodynamic and respiratory status. Cervical examination revealed a painful cervical mass that was mobile during swallowing. Laboratory investigations, including thyroid function tests, were within normal limits. Cervicothoracic CT revealed a plunging and compressive nodular goiter arising from the left thyroid lobe, associated with subcentimeter laterocervical lymphadenopathy. The patient underwent a tracheotomy with surgical biopsy of the thyroid mass.

**Figure 3:**
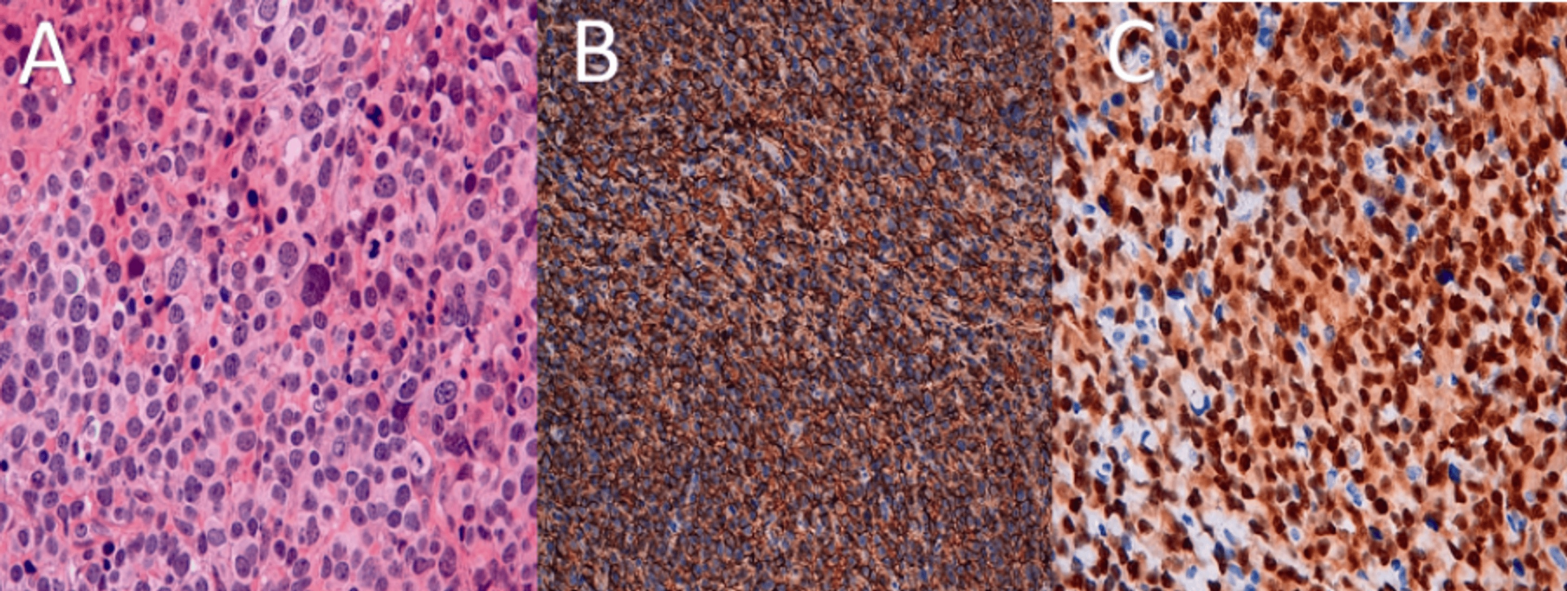
Histopathological and immunohistochemical findings (Case 2) (A) Histopathological section showing large, atypical round cells arranged in diffuse sheets (hematoxylin and eosin stain, ×40). (B) Strong and diffuse CD20 positivity in tumor cells (immunohistochemistry). (C) High tumor proliferation index, estimated at approximately 90% (Ki-67 immunostaining).

Histological examination showed a tumor proliferation arranged in diffuse sheets of large round cells with a high nucleocytoplasmic ratio, eosinophilic cytoplasm, and numerous mitotic figures (Figure 3A). Immunohistochemical analysis demonstrated strong and diffuse CD20 positivity in tumor cells (Figure 3B), a high tumor proliferation index estimated at approximately 90% (Figure 3C), and absence of tumor cell staining for CD3 and CD5. Based on histopathological and immunohistochemical findings, the tumor was diagnosed as diffuse large B-cell lymphoma (DLBCL).

The patient underwent six courses of chemotherapy following the R-CHOP protocol. The mass showed significant regression, but the patient was subsequently lost to follow-up.

Case 3

**Figure 4:**
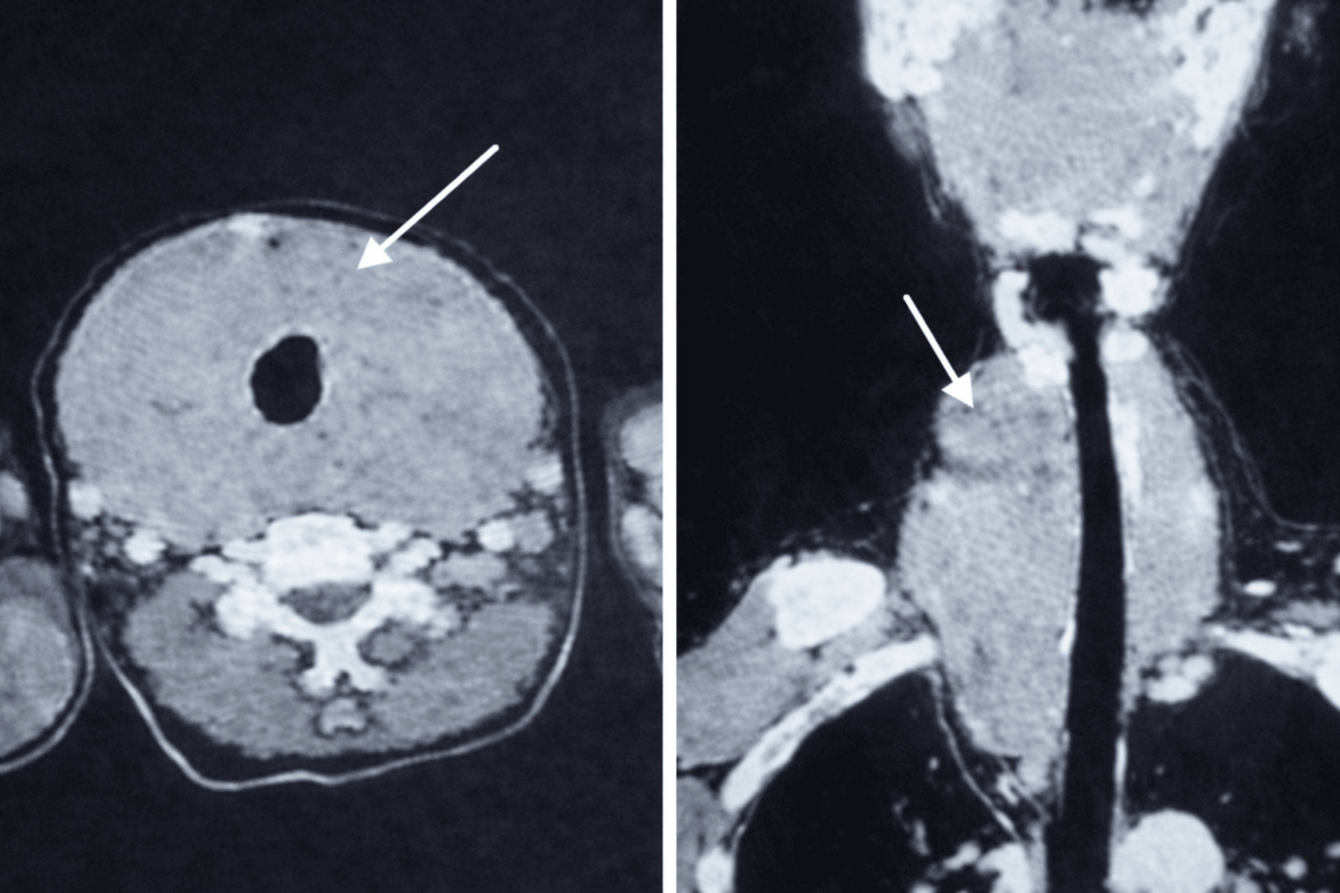
CT images showing a thyroid mass extending into the superior mediastinum and causing deviation of the trachea White arrows indicate the location of the mass.

A 77-year-old woman with no significant past medical history presented with a rapidly enlarging thyroid mass of three weeks' duration, associated with dysphagia and dyspnea. Cervicothoracic CT revealed a circumferential thyroid tumor extending into the suprasternal mediastinum (Figure 4).

**Figure 5:**
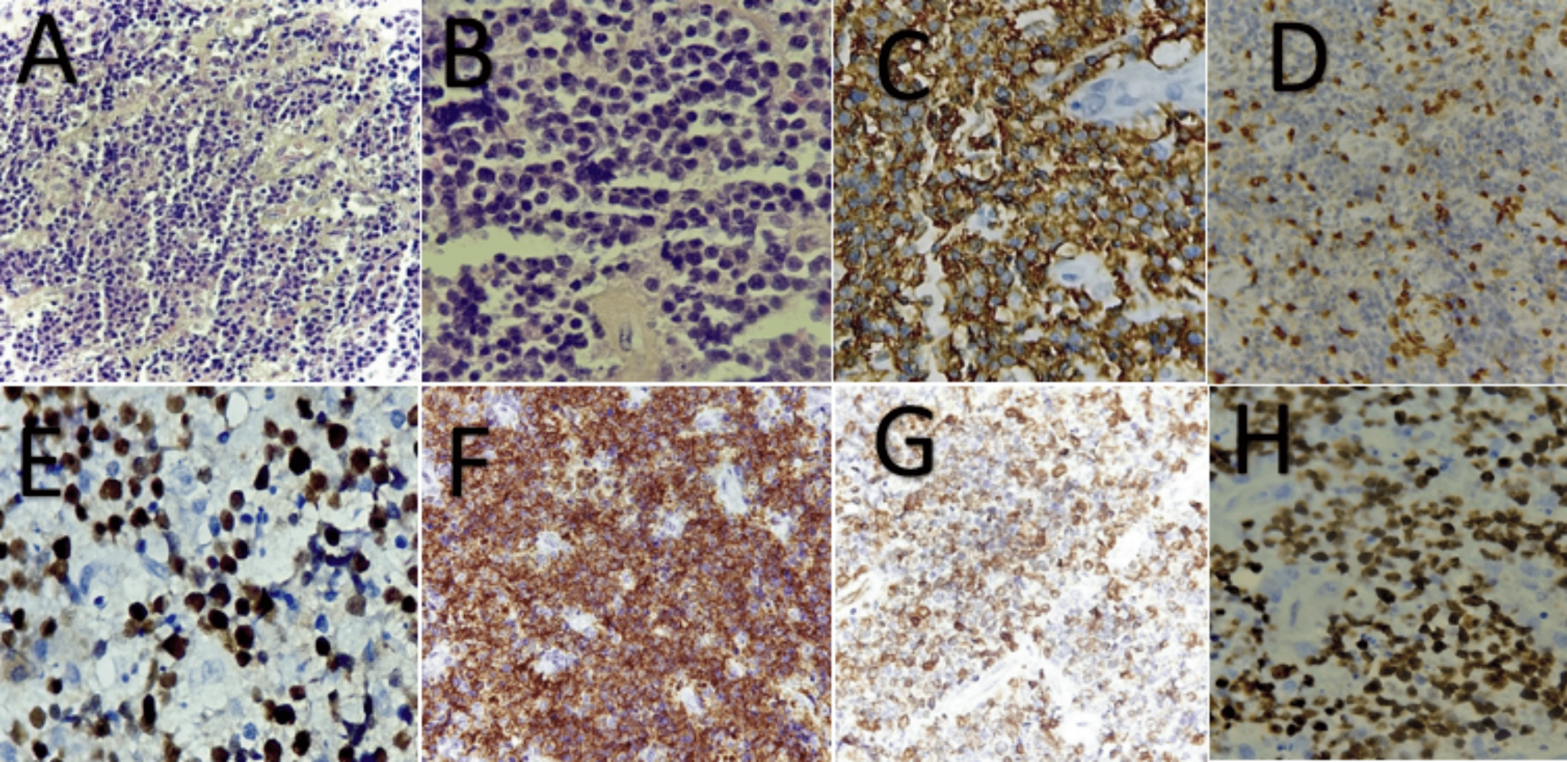
Histopathological and immunohistochemical findings (Case 3) A) Histopathology section showing tumor cells arranged in sheets (hematoxylin and eosin staining, magnification x20). B) Tumor cells appear round, medium to large in size, with several mitotic figures. C) Strong and diffuse positive staining of tumor cells by the anti-CD20 antibody. D) Reactive staining of tumor cells with CD3. E) Positive staining of tumor cells with Bcl6. F) Positive staining of tumor cells with CD10. G) Positive staining of more than 40% of tumor cells with Bcl2. H) The Ki-67 proliferation index is estimated to be 90%.

Histological examination revealed a malignant tumor proliferation arranged in diffuse sheets. The tumor consisted of medium to large atypical cells with hyperchromatic nuclei and clear cytoplasm. Numerous macrophages with tingible bodies and karyorrhectic nuclei were also present, and various mitotic figures were observed (Figures 5A, 5B). Immunohistochemical analysis demonstrated strong positivity of tumor cells for CD20, CD10, and BCL6 (Figures 5C, 5E, 5F), with BCL2 positive in more than 40% of tumor cells (Figure 5G). Tumor cells showed reactive staining for CD3 and CD5 (Figure 5D), whereas staining for cytokeratin (CK), calcitonin, and thyroglobulin was absent. The tumor proliferation index (Ki-67) was estimated at approximately 90% (Figure 5H).

Based on the combined histological and immunohistochemical findings, a diagnosis of high-grade B-cell lymphoma of the thyroid was established.

The patient initiated chemotherapy but experienced rapid clinical deterioration, leading to death shortly thereafter.

## Discussion

Typically, the thyroid gland does not contain native intrathyroidal lymphoid tissue [1]. The presence of lymphoid tissue within the thyroid is most often secondary to chronic antigenic stimulation related to autoimmune thyroid diseases, particularly Hashimoto’s thyroiditis and Graves’ disease [2,3]. Several studies have demonstrated a strong association between immune dysregulation and lymphomagenesis [3]. PTL is closely associated with Hashimoto’s thyroiditis, as reported by Mubarak Al-Mansour et al. [3], and may be identified by elevated antithyroid antibody levels or characteristic histopathological findings. A descriptive epidemiological study has shown that Hashimoto’s thyroiditis increases the risk of PTL by 40- to 80-fold [4].

The pathogenesis of PTL remains incompletely understood. Recent studies have implicated the Wnt5a/Ror2 signaling pathway in the development of PTL [5]. In this context, a Chinese study involving 22 patients diagnosed with PTL evaluated Wnt5a and Ror2 expression using immunohistochemistry with rabbit anti-human Wnt5a and Ror2 antibodies [6]. The authors suggested that Wnt5a may act as a tumor suppressor during the early stages of PTL; however, as the disease progresses, its regulatory role appears to be lost, contributing to uncontrolled tumor growth.

Clinically, PTL typically presents as a painless cervical mass with rapid enlargement, particularly in cases of diffuse large B-cell lymphoma (DLBCL), whereas growth is usually slower in mucosa-associated lymphoid tissue (MALT) lymphoma [7]. Rapid tumor enlargement may lead to compression of adjacent structures, resulting in symptoms such as dyspnea and dysphagia, as observed in our three patients [1]. Additional compressive symptoms include dysphonia, stridor, and cough [4]. Systemic “B symptoms” (fever, weight loss, and night sweats) are reported in approximately 10-20% of patients [4].

Physical examination typically reveals a firm cervical mass, which may be unilateral or bilateral, and cervical lymphadenopathy may be present, as noted in two of our patients [1,7].

Thyroid function tests most often demonstrate euthyroidism [4]. Hypothyroidism is observed in approximately 10% of cases, and anti-thyroperoxidase antibodies are positive in more than 60% of patients, as illustrated by the first patient in our series [1,4].

Neck ultrasonography plays a pivotal role in the initial evaluation of cervical masses [1]. PTL typically appears as a hypoechoic mass with internal hyperechoic linear or reticular areas [8]. Ultrasound findings allow classification of PTL into three patterns: nodular, diffuse, or mixed [9]. This classification is based on internal echogenicity, posterior acoustic features, and lesion margins [10]. In the diffuse type, the mass lacks distinct nodules and may involve part of a thyroid lobe, an entire lobe, or the whole gland [8]. Most studies report well-defined margins, except in cases of Hodgkin lymphoma [8].

Computed tomography (CT) of the neck should be performed once PTL is suspected, as it allows accurate localization of the tumor, assessment of local extension, and staging [10]. CT is particularly useful for detecting lymphadenopathy that may not be visible on ultrasound [10]. PTL typically appears as a soft-tissue density mass that enhances after contrast administration [11]. It may present as a solitary nodule, multiple nodules, or diffuse homogeneous enlargement of the thyroid gland, as observed in our patients [10].

Magnetic resonance imaging (MRI) may help identify residual normal thyroid tissue and detect secondary lesions [10,12]. Currently, fluorine-18 fluorodeoxyglucose positron emission tomography (FDG-PET) is considered a valuable tool for both staging and follow-up [10]. PET/CT, which combines functional and anatomical imaging, further improves diagnostic accuracy and staging precision [10].

When thyroid nodules are detected, fine-needle aspiration (FNA) biopsy is usually the first diagnostic procedure performed [4]. However, several studies have demonstrated the limited specificity of FNA in diagnosing PTL. Comparative analyses indicate that core needle biopsy provides superior diagnostic accuracy and allows better lymphoma subtyping [13]. Even when FNA suggests PTL, additional tissue sampling is often required [3]. One study recommended core needle biopsy as the preferred initial diagnostic approach when PTL is suspected [14].

The use of ultrasound guidance and ancillary techniques, such as flow cytometry, immunohistochemistry, and molecular studies, particularly polymerase chain reaction, has improved the diagnostic performance of FNA, especially in cases of DLBCL [2,4]. Cytologically, the main differential diagnosis of DLBCL is anaplastic large-cell lymphoma. DLBCL is characterized by a population of discohesive, pleomorphic large cells with lymphoglandular bodies, whereas anaplastic lymphoma typically shows cell clustering, nuclear molding, and absence of lymphoglandular bodies [4]. Distinguishing MALT lymphoma from Hashimoto’s thyroiditis remains particularly challenging due to overlapping cytological features [2].

Surgical biopsy is reserved for cases in which minimally invasive procedures fail to establish a definitive diagnosis or accurately classify the lymphoma subtype [10]. It provides sufficient tissue for comprehensive histopathological and immunohistochemical evaluation and may be required in selected cases [1].

Histologically, DLBCL is characterized by a diffuse proliferation of large atypical lymphoid cells that disrupt thyroid follicles. Tumor cells display vesicular nuclei and moderate amphophilic cytoplasm [15]. Immunohistochemically, they are typically positive for CD20, Bcl-6 in approximately 75% of cases, and Bcl-2 in about 50% of cases [16]. DLBCL of the thyroid is associated with a relatively poor prognosis and is subdivided into germinal center B-cell-like and activated B-cell-like subtypes, the former generally exhibiting a more favorable outcome [16].

MALT lymphoma is characterized by lymphoepithelial lesions, in which neoplastic lymphoid cells infiltrate and colonize thyroid follicles. These lesions are highlighted by CD20 and cytokeratin immunostaining [15,16]. Tumor cells are typically negative for CD5, CD10, and CD23 [16]. Although MALT lymphoma is generally indolent, transformation into an aggressive lymphoma may occur over time [16].

Follicular lymphoma of the thyroid is defined by a destructive proliferation of atypical lymphoid cells forming a follicular architecture, often accompanied by an interfollicular infiltrate of neoplastic B cells [17]. Lymphoepithelial lesions are frequently observed [15]. Immunohistochemistry demonstrates expression of germinal center markers (Bcl-6 and/or CD10), commonly associated with Bcl-2 positivity [16,17]. Thyroid follicular lymphomas can be divided into two groups. The first group shows IGH/BCL2 translocation and/or BCL2 overexpression and is usually of lower histological grade but more frequently presents with extrathyroidal disease [14,16,18]. The second group lacks IGH/BCL2 rearrangement and BCL2 expression, is generally of higher grade, but less often associated with extrathyroidal involvement, and appears to have a better prognosis [16].

Regarding treatment, localized PTL confined to the thyroid gland is primarily managed with cervico-mediastinal radiotherapy, achieving a 5-year survival rate of approximately 90% [3,18]. Combined chemotherapy based on the R-CHOP regimen and radiotherapy is recommended in cases with extracapsular extension or advanced disease [19]. Surgery is currently limited to diagnostic purposes or, in selected cases, to relieve compressive symptoms [3,20].

## Conclusions

In conclusion, primary thyroid lymphoma is a rare tumor that is strongly associated with Hashimoto's thyroiditis. In a woman with lymphocytic thyroiditis who presents with a rapidly enlarging thyroid mass and compressive symptoms, PLT should be considered as the primary diagnosis. Core needle biopsy and incisional biopsy are the preferred techniques for obtaining sufficient material to confirm the diagnosis and determine the histological subtype. Management is multidisciplinary, and prognosis depends on both the lymphoma subtype and disease stage.
